# An *SCN1B* Variant Affects Both Cardiac-Type (Na_V_1.5) and Brain-Type (Na_V_1.1) Sodium Currents and Contributes to Complex Concomitant Brain and Cardiac Disorders

**DOI:** 10.3389/fcell.2020.528742

**Published:** 2020-09-29

**Authors:** Rebecca Martinez-Moreno, Elisabet Selga, Helena Riuró, David Carreras, Mered Parnes, Chandra Srinivasan, Michael F. Wangler, Guillermo J. Pérez, Fabiana S. Scornik, Ramon Brugada

**Affiliations:** ^1^Departament de Ciències Mèdiques, Facultat de Medicina, Universitat de Girona, Girona, Spain; ^2^Cardiovascular Genetics Center, Institut d’Investigació Biomèdica de Girona Dr. Josep Trueta, Girona, Spain; ^3^Faculty of Medicine, University of Vic-Central University of Catalonia (UVic-UCC), Vic, Spain; ^4^Centro de Investigación Biomédica en Red de Enfermedades Cardiovasculares (CIBERCV) Madrid, Spain; ^5^Blue Bird Circle Clinic for Pediatric Neurology, Section, of Pediatric Neurology and Developmental Neuroscience, Texas Children’s Hospital, Baylor College of Medicine, Houston, TX, United States; ^6^Section of Pediatric Cardiac Electrophysiology, Division of Pediatric Cardiology, Department of Pediatrics, University of Texas Health Science Center at Houston, Houston, TX, United States; ^7^Texas Children’s Hospital, Houston, TX, United States; ^8^Department of Molecular and Human Genetics, Baylor College of Medicine, Houston, TX, United States; ^9^Hospital Josep Trueta, Girona, Spain

**Keywords:** Na_V_1.5, Na_V_1.1, Na_V_β1, Na_V_β1b, cardiac arrhythmia, brain hyperexcitability

## Abstract

Voltage-gated sodium (Na_V_) channels are transmembrane proteins that initiate and propagate neuronal and cardiac action potentials. Na_V_ channel β subunits have been widely studied due to their modulatory role. Mice null for *Scn1b*, which encodes Na_V_ β1 and β1b subunits, have defects in neuronal development and excitability, spontaneous generalized seizures, cardiac arrhythmias, and early mortality. A mutation in exon 3 of *SCN1B*, c.308A>T leading to β1_p.D103V and β1b_p.D103V, was previously found in a patient with a history of proarrhythmic conditions with progressive atrial standstill as well as cognitive and motor deficits accompanying structural brain abnormalities. We investigated whether β1 or β1b subunits carrying this mutation affect Na_V_1.5 and/or Na_V_1.1 currents using a whole cell patch-clamp technique in tsA201 cells. We observed a decrease in sodium current density in cells co-expressing Na_V_1.5 or Na_V_1.1 and β1^D103V^ compared to β1^WT^. Interestingly, β1b^D103V^ did not affect Na_V_1.1 sodium current density but induced a positive shift in the voltage dependence of inactivation and a faster recovery from inactivation compared to β1b^WT^. The β1b^D103V^ isoform did not affect Na_V_1.5 current properties. Although the *SCN1B*_c.308A>T mutation may not be the sole cause of the patient’s symptoms, we observed a clear loss of function in both cardiac and brain sodium channels. Our results suggest that the mutant β1 and β1b subunits play a fundamental role in the observed electrical dysfunction.

## Introduction

Voltage-gated sodium (Na_V_) channels are transmembrane proteins that initiate and propagate neuronal and cardiac action potentials (Catterall, 2000; Catterall et al., 2005). These channels have an α subunit composed of four domains (DI–DIV), each with six transmembrane segments (S1–S6). Na_V_α subunits are associated with two or more auxiliary β subunits (Kaplan et al., 2016). Na_V_β subunits modulate sodium channel biophysical properties in excitable and non-excitable tissues and function as cell adhesion molecules, which are critical for extracellular/intracellular communication (Chen, 2004; Watanabe et al., 2008; Patino et al., 2011).

Five Na_V_β subunits have been identified in mammals: β1, β2, β3, β4 (encoded by *SCN1B*, *SCN2B*, *SCN3B*, and *SCN4B*, respectively), and β1b, a β1 splice variant. All β subunits with the exception of β1b are composed of an extracellular N-terminal immunoglobulin-like (Ig) domain (ECD), a transmembrane region, and an intracellular C-terminal domain (Brackenbury and Isom, 2011). Similar to the other β subunits, β1b is composed of an N-terminal region encoded by exons 1–3 of *SCN1B*. However, instead of a transmembrane domain, β1b has a different C-terminal region caused by partial retention of intron 3, which contains a stop codon (Hu et al., 2012). Apart from its structural differences, β1b is the only extracellularly secreted β subunit (Patino et al., 2011; O’Malley and Isom, 2016). However, it is predicted to have the same cellular interactions as the β1 subunit because it shares the same ECD (O’Malley and Isom, 2016).

In a series of experiments using Na_V_β subunit chimeras, McCormick et al. (1999) demonstrated that the ECD is necessary and sufficient for sodium channel modulation (McCormick et al., 1999). Mutations in the ECD of the β1 subunit have been associated with brain diseases, such as epileptic encephalopathy and genetic (or generalized) epilepsy with febrile seizures plus (Wallace et al., 1998; Audenaert et al., 2003; Scheffer et al., 2007; Brackenbury and Isom, 2011; Darras et al., 2019). Some of these mutations have been proven to disrupt the interaction between Na_V_1.1 and the β1 subunit, impairing excitability or reducing cell-cell interactions (Meadows et al., 2002; Chen, 2004; Spampanato, 2004; Patino and Isom, 2010). Although Na_V_α subunit alone is sufficient to form a conducting channel, β1 subunits enhance protein expression and modulate gating and voltage dependency (McCormick et al., 1999; Meadows et al., 2002; Yu and Catterall, 2003; Olesen et al., 2012). *Scn1b*-null mice have defective neuronal development and excitability, present spontaneous generalized seizures and cardiac arrhythmias, and die by postnatal day 21 (Chen, 2004; Patino and Isom, 2010; Brackenbury and Isom, 2011; Lin et al., 2015).

The regulatory effect of the β1^WT^ subunit on Na_V_1.5 has been widely studied. *SCN1B* mutations are reported to cause diseases such as Brugada syndrome (Watanabe et al., 2008; Peeters et al., 2015), long QT syndrome (Riuró et al., 2014), and atrial fibrillation (Olesen et al., 2012), demonstrating the critical effect of β1 on cardiac excitability. In addition, Lopez-Santiago et al. (2007) showed that *Scn1b*-null mice display prolonged QT and RR intervals. In addition, mutations in β1b have been associated with cardiac arrhythmias, such as Brugada syndrome, long QT syndrome, cardiac conduction disease, and SIDS (Watanabe et al., 2008; Hu et al., 2012; Riuró et al., 2014).

We investigated a mutation located in exon 3 of *SCN1B* [Chr19:35524503 A>T (hg19): NM_001037.4 c.308A>T] identified in a patient with a history of proarrhythmic conditions with progressive atrial standstill, cognitive and motor deficits, and structural brain abnormalities. The patient was part of a sequencing study by Eldomery et al. (2017) that proposed a dual diagnosis for this case. As a result, this β1 variant (β1^D103V^) (dbSNP ID: 1057519457) is indexed as pathogenic in the ClinVar database^1^. However, this variant has not been functionally characterized.

The mutation leads to substitution of an aspartic acid for a valine at position 103 of the protein (p.D103V). Exon 3 is part of the Ig-like loop of the ECD, which is common between β1 and β1b. Therefore, this substitution is present in both β1 and β1b subunit isoforms at a highly conserved position. Thus, this mutation likely affects the function of both β1 and β1b proteins, and could play a role in the patient’s pathophysiologic phenotype. This work characterizes the effects of mutant β1 and β1b subunits on the biophysical properties of both cardiac (Na_V_1.5) and neuronal (Na_V_1.1) sodium channels.

## Materials and Methods

### Expression Vectors and Site-Directed Mutagenesis

The pCMV vector harboring *SCN1A* was a generous gift from Dr. Alfred George Jr. (Vanderbilt University Division of Genetic Medicine, Nashville, TN, United States). The pcDNA3 vector harboring the complementary DNA (cDNA) of human *SCN5A* was a generous gift from Dr. Matteo Vatta (Baylor College of Medicine, Houston, TX, United States).

Commercially available human *SCN1B* cDNA (pCMV6-XL4-SCN1B OriGene Technologies Inc., Rockland, MD, United States) was subcloned into a bicistronic vector encoding enhanced green fluorescent protein (GFP) (pIRES-GFP, Clontech Laboratories Inc., Mountain View, CA, United States). We constructed a bicistronic vector encoding SCN1Bb-GFP (pIRES-GFP-SCN1Bb). Human *SCN1Bb* cDNA was cloned from the human right ventricle (Riuró et al., 2014).

pIRES-GFP-SCN1B and pIRES-GFP-SCN1Bb were used as templates to engineer the p.D103V mutation using a QuikChange Lightning site-directed mutagenesis kit (Stratagene, La Jolla, CA, United States) and the following primers (mutation underlined):

Forward: 5′ CACCAAAGACCTGCAGGTTCTGTCTATCTT CATCA 3′.

Reverse: 5′ TGATGAAGATAGACAGAACCTGCAGGTCTT TGGTG 3′.

The inserts were sequenced to verify presence of the desired mutation and absence of unwanted variations.

### Cell Culture and Transfection

Human embryonic kidney (HEK)-293 tsA201 cells (Health Protection Agency Culture Collections, Salisbury, United Kingdom) were used to express the sodium channel subunits. Cells were maintained at 37°C and 5% CO_2_ in Dulbecco’s Modified Eagle Medium supplemented with 10% fetal bovine serum, 1% penicillin-streptomycin, and 1% GlutaMAX (all from Gibco, Thermo Fisher Scientific Inc., Waltham, MA, United States). HEK cells were plated on 35 mm dishes coated with poly-L-lysine (Sigma-Aldrich Co., St. Louis, MO, United States). Cells were transiently transfected 24 h after plating using Lipofectamine 2000 (Invitrogen, Thermo Fisher Scientific Inc.) and Opti-MEM (Gibco, Thermo Fisher Scientific Inc.) with 2 μg of total DNA encoding Na_V_1.5 alone, Na_V_1.5+β1^WT^, Na_V_1.5+β1^D103V^, Na_V_1.1 alone, Na_V_1.1+β1^WT^, Na_V_1.1+β1^D103V^, Na_V_1.1+β1b^WT^, or Na_V_1.1+β1b^D103V^ at a 1:2 (α:β) molar ratio. The effect of the β1b subunit on Na_V_1.5 current was studied by transfecting Na_V_1.5 alone, Na_V_1.5+β1b^WT^, or Na_V_1.5+β1b^D103V^ at a 1:2 molar ratio using a Genecellin transfection kit (BioCellChallenge, Toulon, France). HEK cells were split and re-plated 24 h after transfection to obtain single cells. Electrophysiological studies were performed 48 h after transfection.

### Electrophysiological Studies

Sodium currents from cells displaying green fluorescence were studied at room temperature using the patch clamp whole cell configuration. The bath solution contained (in mmol/l): 140 NaCl, 3 KCl, 10 N-2-hydroxyethylpiperazine-N′-2-ethanesulfonic acid (HEPES), 1.8 CaCl_2_ and 1.2 MgCl_2_, pH 7.4 (NaOH). The pipette solution contained (in mmol/l): 130 CsCl, 1 ethylene glycol-bis(2-amino-ethylether)-N,N,N′,N′-tetra-acetic acid (EGTA), 10 HEPES, 10 NaCl and 2 ATP-Mg^2+^, pH 7.2 (CsOH). Osmolality of bath and pipette solutions was adjusted with glucose to 325 and 308 mOsm, respectively. Pipettes were pulled from borosilicate glass capillaries (Sutter Instrument, Novato, CA, United States) with a Narishige PC-10 puller (Narishige International LTD, London, United Kingdom), and their resistance ranged 2–3.5 MΩ when filled with pipette solution. Voltage clamp experiments were conducted with an Axopatch 200B amplifier and pClamp10.2/Digidata 1440A acquisition system (Axon Instruments, Molecular Devices, Sunnyvale, CA, United States) at a sampling rate of 20 kHz. Leak subtraction was not used. OriginPro8 software was used for data analysis and statistics (OriginLab Corporation, Northampton, MA, United States). Data was filtered at 5 kHz. Series resistance compensation of 80–90% was used when necessary. To permit current stabilization, recordings were performed at least 5 min after entering into the whole cell configuration. Membrane potentials were not corrected for junction potentials that arose between the bath and pipette solution.

Sodium currents were studied with voltage-clamp step protocols. To determine the current-voltage relationship and voltage dependence of activation, cells were held at –120 mV and currents were elicited with depolarizing pulses of 50 ms from –80 to 80 mV in 5 mV increments. The voltage dependence of inactivation was determined by applying 50-ms prepulses from –140 to 5 mV in 5 mV increments, followed by a –20 mV test pulse. Recovery from inactivation was studied with a two-pulse protocol consisting of a first –20 mV pulse of 50 ms from a holding potential of –120 mV, followed by a –120 mV interpulse of varying duration (1–40 ms), and a second –20 mV pulse of 50 ms. Current density was obtained by normalizing the current at each potential by cell capacitance. Activation and steady-state inactivation data were fitted to a Boltzmann equation of the form *G* = *G*_max_/(1+exp(*V*_1/2_–*V*)/k) and *I* = *I*_max_/(1+exp(*V*–*V*_1/2_)/k), respectively, where G is conductance, Gmax is maximum conductance, *V*_1/2_ is voltage at which half of channels are activated or inactivated, *V* is membrane potential, *k* is slope factor, *I* is peak current amplitude, and *I*_max_ is maximum current amplitude. Recovery from inactivation data were fitted to a mono-exponential function to obtain the time constant.

### Western Blot

Cells were plated and transfected as above. Forty-eight hours after transfection, cells were washed three times with Dulbecco’s phosphate-buffered saline (DPBS) and scraped in Triton X-100 lysis buffer containing 1% Triton X-100, 50 mM Tris/HCl pH 7.4, 150 mM NaCl, 1 mM EDTA and cOmplete protease inhibitor cocktail (Roche, Madrid, Spain). Lysates were obtained after 1 h rotating at 4°C, and insoluble materials were removed by centrifugation. Proteins were quantified using a Pierce BCA protein assay kit (Thermo Scientific, Rockford, IL, United States) and resolved along with a protein marker (PageRuler Plus prestained protein ladder, Thermo Scientific) in 4–15% Mini-PROTEAN TGX Stain-Free precast gels (Bio-Rad Laboratories, Hercules, CA, United States). These gels include a trihalo compound that react with tryptophan residues in proteins, and allow rapid fluorescent detection of proteins in gels or on membranes without staining. Proteins were transferred to PVDF membranes (GE Healthcare Life Sciences, Chicago, IL, United States) overnight at 4°C. Gels were developed by exposure to UV light before and after protein transfer to the membrane. Membranes were probed with either a rabbit anti-human Na_V_1.5 antibody (Alomone Labs) at a 1:1000 dilution for 1 h at room temperature, or a rabbit anti-human Na_V_1.1 antibody (Alomone Labs, Jerusalem, Israel) at a 1:100 dilution overnight at 4°C. Membranes were further incubated with a secondary horseradish peroxidase-conjugated anti-rabbit antibody (Thermo Scientific) at a 1:10000 dilution for 1 h at room temperature. Chemiluminescent signal was obtained with Pierce ECL western blotting substrate (Thermo Scientific) and detected using standard X-ray films. Expression of Na_V_1.1 and Na_V_1.5 was quantified from digitized X-ray film images using ImageJ software (National Institutes of Health,^2^). Membrane intensity values for each sample were normalized by total lane density before protein transfer (Taylor et al., 2013).

### Cell Surface Protein Biotinylation

Cell surface protein biotinilation was performed following the protocol from Tarradas et al. (2013). Briefly, cells were plated and transfected as described above. Twenty-four hours after transfection, cells were washed twice with DPBS supplemented with 0.9 mM CaCl_2_ and 0.49 mM MgCl_2_ (DPBS^+^). Membrane proteins were biotinylated by incubating cells with 1 mg/ml of EZ-Link Sulfo-NHS-SS-Biotin (Thermo Scientific) in DPBS^+^ for 30 min at 4°C. Cells were then washed three times in DPBS^+^ with 100 mM glycine, and scrapped in Triton X-100 lysis buffer [1% Triton X-100, 50 mM Tris/HCl pH 7.4, 150 mM NaCl, 1 mM EDTA and Complete Protease Inhibitor Cocktail (Roche, Madrid, Spain)]. Lysates were obtained after 1 h rotating at 4°C. Insoluble materials were removed by centrifugation. 10% of the supernatants were kept at −80°C (input samples), the rest (pull-down samples) were incubated with Ultralink Immobilized NeutrAvidin resin (Pierce, Thermo Scientific) overnight at 4°C. The resin was precipitated and washed with Triton X-100 lysis buffer, then in saline solution (5 mM EDTA, 350 mM NaCl and 0.1% TX-100 in DPBS^+^) and finally in 50 mM Tris/HCl pH 7.4, 150 mM NaCl and 1 mM EDTA. Input and pull-down samples were resuspended in SDS-PAGE loading buffer and heated for 5 min at 95°C. Proteins were resolved in 7.5% acrylamide gels using TGX Stain-Free FastCast Acrylamide Kit (Bio-Rad Laboratories) and transferred overnight at 4°C to PVDF membranes (Millipore, Billerica, MA, United States). Protein bands using Stain-Free gels were visualized by exposure to UV light before electroblotting to PVDF membranes. Membranes were probed with a rabbit anti-human Na_V_1.5 antibody (Alomone Labs) at a 1:1000 dilution either for 1 h at room temperature or overnight at 4°C. After washing, the membrane was further incubated with a secondary horseradish peroxidase-conjugated antibody (Thermo Scientific, Rockford, IL, United States) at a dilution of 1:10000 for 1 h at room temperature. Chemiluminescent signal was obtained with Clarity Western ECL Substrate (Bio-Rad Laboratories) and detected using the ChemiDoc Imaging System (Bio-Rad Laboratories). Expression of Na_V_1.5 was quantified using ImageJ software (National Institutes of Health,^3^). Membrane intensity values for each sample were normalized by total lane density before protein transfer (Taylor et al., 2013).

### Statistical Analisys

OriginPro 2019 software was used for statistical analysis. Results are presented as means ± standard error. Statistical comparisons were performed using one-way ANOVA with a post hoc Tukey test. Differences were considered statistically significant at *p* < 0.05.

## Results

### An *SCN1B* Mutation Found by Whole Exome Analysis Is Linked to Cardiac and Brain Dysfunction

**FIGURE 1:**
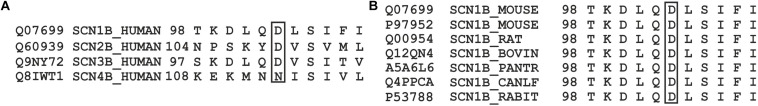
The protein region that includes the mutation is highly conserved. Sequence alignments of the region shared by β1 and β1b proteins containing the D103V mutation were performed using Uniprot. The position of the last amino acid of each sequence, and the reference for each protein according to Uniprot are indicated on the left side of each sequence. **(A)** Sequence alignment of human voltage-gated sodium channel β-subunit family members. **(B)** Sequence alignment of human voltage-gated sodium channel β1-subunit of different species.

The proband, currently an 8-year-old male, was part of a whole exome sequencing study by Eldomery et al. (2017). This study revealed that the proband was heterozygous for the *SCN1B* missense mutation c.308A>T. This mutation results in an amino acid change from a hydrophilic aspartic acid to a hydrophobic valine at position 103 (p.D103V) of the sodium channel auxiliary β subunit. D103 is probably important to channel function, as it is highly conserved among all sodium channel β subunits, except for β4, and is also conserved among different species (Figures 1A,B). Eldomery et al. indexed the β1 subunit variant D103V as pathogenic (Eldomery et al., 2017).

In addition to the *SCN1B* variant, inherited from the child’s father, genetic analysis revealed that the proband was heterozygous for two pathogenic variants in *POLR1C*: c.614delG (p.G205Afs^∗^49), for which his mother was heterozygous, and c.88C>T (p.P30S), for which his father was heterozygous.

**FIGURE 2:**
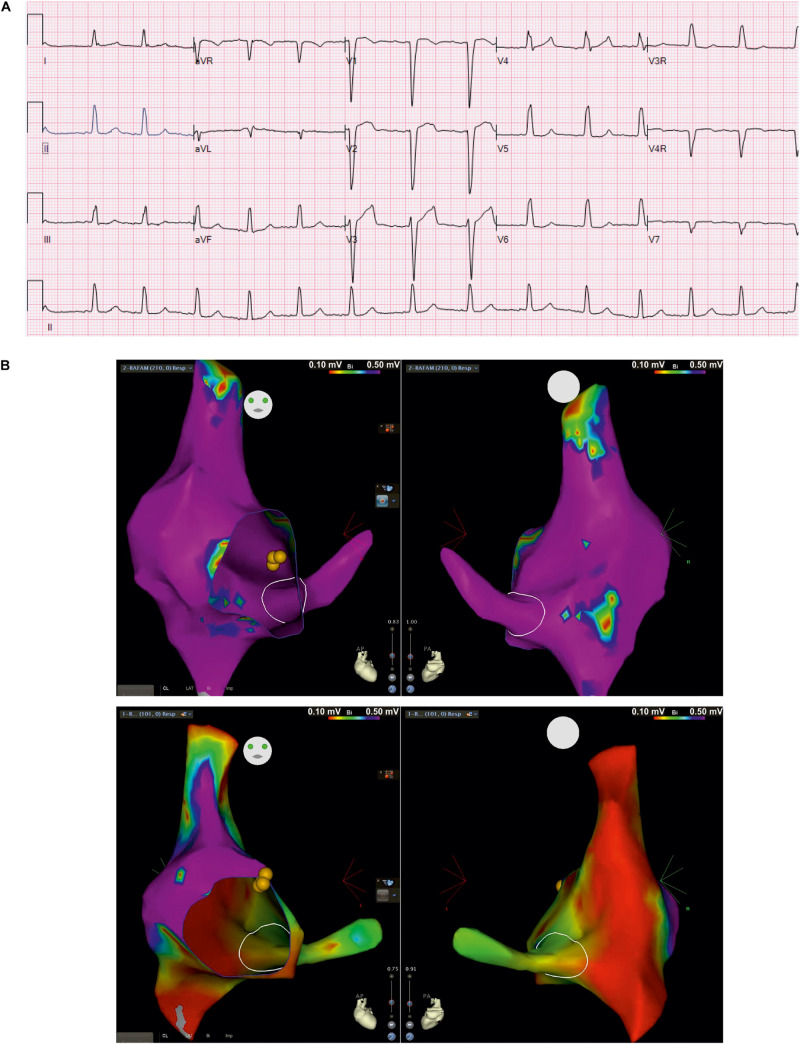
Surface electrocardiogram and intracardiac three-dimensional voltage mapping. **(A)** Resting surface electrocardiograms (ECG) at 5 years of age in index patient at 25 mm/second, 10 mm/mV; **(B)** Antero-posterior (left) and postero-anterior (right) projections of voltage map of right atrium and coronary sinus in baseline rhythm from index patient (top) and, healthy age-matched peer (bottom) for comparison (voltage color scale range: 0.1 – 0.5 mV, “purple” indicates voltage >0.5 mV)

The clinical picture of the patient is complex and includes cardiac and neurological impairment. Arrhythmogenic features presented early in the neonatal stage. These included bradycardia from variable atrioventricular (AV) conduction disturbance along with evidence of a borderline prolongation of QTc interval, which appeared to be secondary to intraventricular conduction delay. This was treated with propranolol. Ventricular dysfunction was observed during the neonatal period but normalized at 18 months of age with medical management. Follow-up evaluations showed poorly discernible atrial activity and intraventricular conduction delays on surface electrocardiograms (ECG). The child developed recalcitrant tachycardia at 7 years of age, identified as cavo-tricuspid isthmus (CTI)-dependent macro-reentrant atrial tachycardia (cycle length ∼420 ms) with 1:1 AV conduction as well as 2:1 AV conduction during intracardiac electrophysiology study. At baseline, he was variably in junctional rhythm with brief periods of atrially mediated rhythm. Normal AV conduction was seen at baseline, with atrial pacing that worsened to 2:1 AV conduction on isoproterenol. Three-dimensional voltage mapping at baseline rhythm revealed extensive scarring of the right atrium and coronary sinus. Resting surface electrocardiogram from index patient (Figure 2A) shows poorly discernible atrial activity with low amplitude P wave, intraventricular conduction delay (QRS duration 114 ms) as well as prolonged QTc secondary to QRS abnormality (uncorrected QTc 477 ms). Intracardiac three-dimensional voltage map from index patient is contrasted against a healthy age-matched peer (Figure 2B). The patient underwent placement of a transvenous dual-chamber pacemaker. He was programmed in the VVI pacing mode due to progressive atrial undersensing and unreliable atrial capture.

By the 2 year of life, the patient also had global developmental delay and increased tone in the lower extremities. Neurological examination at age five demonstrated evidence of significant cognitive deficits (smiled socially, followed simple commands, gave one-word answers to questions), persistent large-amplitude horizontal nystagmus with primary gaze, normal tone in the upper extremities with mixed spasticity and dystonia in the lower extremities (spastic catches and intermittent dystonic extension and inward rotation of the lower extremities when excited). He demonstrated symmetrically brisk deep tendon reflexes with Babinski and Rossolimo signs, and significant dysmetria on the finger-to-nose test as well as axial ataxia affecting the neck and trunk. He sat with support and bore weight with assistance, with scissoring of the legs, and did not ambulate. Brain MRI revealed extensive polymicrogyria involving the bilateral cerebral hemispheres, extensive T2 hyperintense signal throughout the supratentorial white matter with less in the cerebellar white matter and dorsal brainstem, and markedly delayed myelination.

To date, no functional studies of the *SCN1B* mutation D103V have been published. Because this *SCN1B* variant was flagged as the best candidate for electrical dysfunction observed in the patient, we comprehensively analyzed the effects of this variant on heart and brain sodium currents.

### Na_V_1.5 Current Properties Are Modified by β1^WT^ and β1b^WT^ Subunits

**FIGURE 3:**
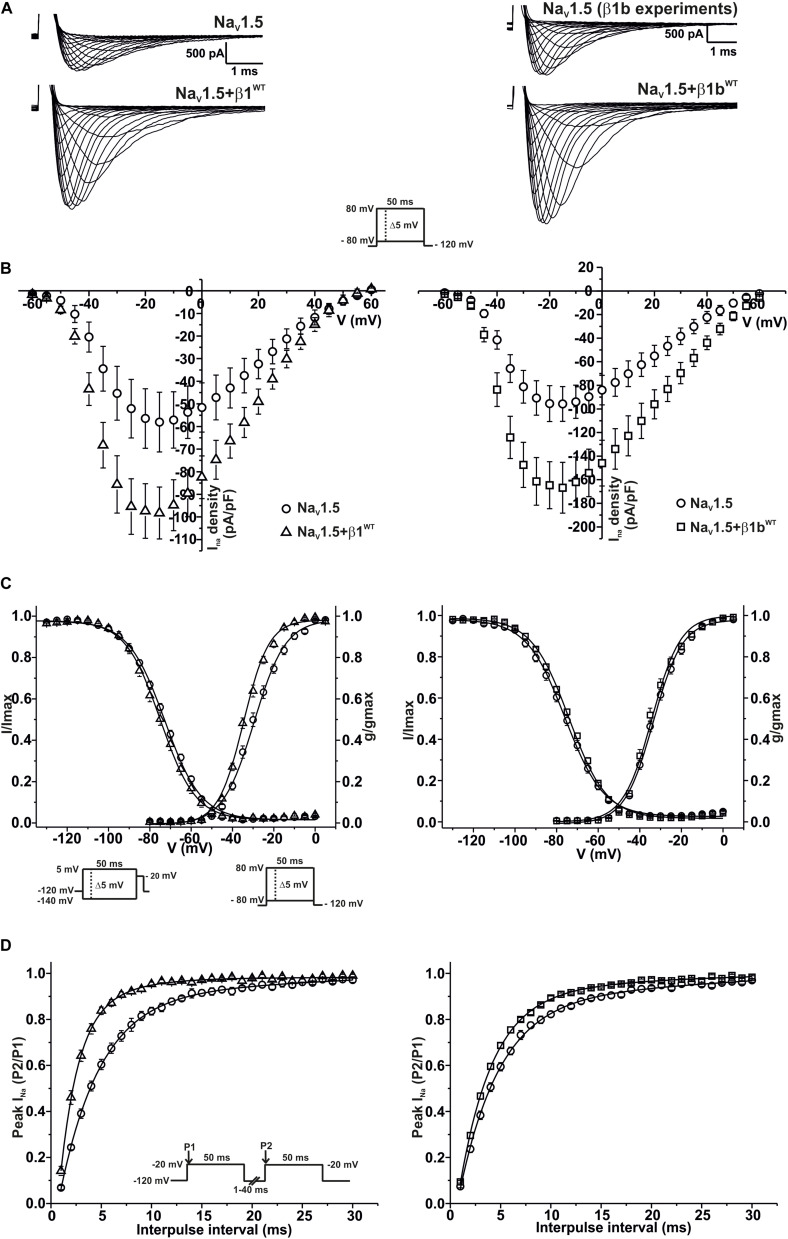
β1^WT^ and β1b^WT^ modulate Na_V_1.5 current. Biophysical properties of sodium currents measured from HEK-293T cells expressing Na_V_1.5 alone, or co-expressed with either β1^WT^ or β1b^WT^. Whole cell currents were elicited by depolarizing potentials as shown in the insets. Circles are used to depict data for Na_V_1.5 alone, triangles for Na_V_1.5+β1^WT^, and squares for Na_V_1.5+β1b^WT^. Solid lines in panels **(C)** and **(D)** represent the fitted curves. Values are expressed as mean ± SEM. The studies with the β1 subunit are represented on the left side of the figure, and the studies with the β1b subunit on the right side. **(A)** Representative whole-cell Na^+^ current traces from HEK-293T cells expressing Na_V_1.5 alone (top left and top right), Na_V_1.5+β1^WT^ (bottom left), and Na_V_1.5+β1b^WT^ (bottom right). **(B)** Mean current-voltage relationship. *I*_Na_ amplitude was normalized by the cell capacitance to obtain *I*_Na_ density values. **(C)** *I*_Na_ steady-state voltage dependence of activation and inactivation plots. **(D)** Recovery from inactivation curves.

Modulation of sodium channels by β subunits has been reported by different groups (Brackenbury and Isom, 2011; O’Malley and Isom, 2016). Our goal was to determine whether mutant β1 (β1^D103V^) and β1b (β1b^D103V^) subunits affected Na_V_1.5 and Na_V_1.1 sodium currents (*I*_Na_). We first performed functional characterization of the sodium current in HEK-293T cells expressing Na_V_1.5 alone or co-expressed with either β1^WT^ (Na_V_1.5+β1^WT^) or β1b^WT^ (Na_V_1.5+β1b^WT^) subunits (Figure 3A). Co-expression of Na_V_1.5 with either β1^WT^ or β1b^WT^ increased peak *I*_*Na*_ density (41.2 and 42.8%, respectively) compared to Na_V_1.5 alone (Figure 3B and Tables 1, 2). Also, the β1^WT^ subunit induced a negative shift of 5 mV in the voltage dependence of activation compared to Na_V_1.5 alone (Figure 3C left and Table 1). No changes were observed in voltage dependence of activation upon expression of Na_V_1.5+β1b^WT^ (Figure 3C right and Table 2). Voltage dependence of inactivation was not altered by either β1^WT^ or β1b^WT^ subunits. Both Na_V_1.5+β1^WT^ and Na_V_1.5+β1b^WT^ currents displayed faster recovery from inactivation compared to Na_V_1.5 alone (Figure 3D and Tables 1, 2).

**TABLE 1:** Biophysical parameters of HEK cells expressing Na_V_1.5 alone or together with β1^WT^.

	Peak I_Na_ density		Activation			Steady-state Inactivation			Recovery	
	pA/pF	*n*	V_1/2_ (mV)	*k*	*n*	*V*_1/2_ (mV)	*k*	*n*	τ (ms)	*n*
Na_V_1.5 alone	−58.51 ± 13.02	8	−29.61 ± 1.10	6.95 ± 0.06	6	−73.00 ± 0.75	9.07 ± 0.21	6	4.42 ± 0.28	6
Na_V_1.5+β1^WT^	−99.50 ± 12.05*	13	−34.61 ± 0.68*	6.40 ± 0.14	11	−74.90 ± 1.33	8.57 ± 0.25	11	2.27 ± 0.16*	10

Data is presented as Mean ± SE. I_Na_ = sodium current; n = number of cells; k = slope factor; V_1/2_ = voltage for half-maximal activation or steady-state inactivation; τ = time constant. *vs Na_V_1.5. Significantly different, p-value < 0.05.

**TABLE 2:** Biophysical parameters of HEK cells expressing Na_V_1.5 alone or together with β1b^WT^.

	Peak *I*_Na_ density		Activation			Steady-state inactivation			Recovery	
	pA/pF	*n*	*V*_1/2_ (mV)	*k*	*n*	*V*_1/2_ (mV)	*k*	*n*	τ (ms)	*n*
Na_V_1.5 alone	−97.44 ± 14.91	15	−33.20 ± 0.88	6.80 ± 0.26	15	−75.69 ± 1.12	9.32 ± 0.20	14	4.56 ± 0.19	12
Na_V_1.5+β1b^WT^	−170.41 ± 22.22*	20	−34.69 ± 0.89	6.14 ± 0.28	20	−73.71 ± 0.62	9.25 ± 0.21	18	3.72 ± 0.11*	10

*Data is presented as Mean ± SE. I_Na_ = sodium current; n = number of cells; k = slope factor; V_1/2_ = voltage for half-maximal activation or steady-state inactivation; τ = time constant. ^∗^vs Na_V_1.5. Significantly different, p-value < 0.05.*

### The Mutant β1^D103V^ Subunit Decreases Na_V_1.5 Sodium Current Density

**FIGURE 4:**
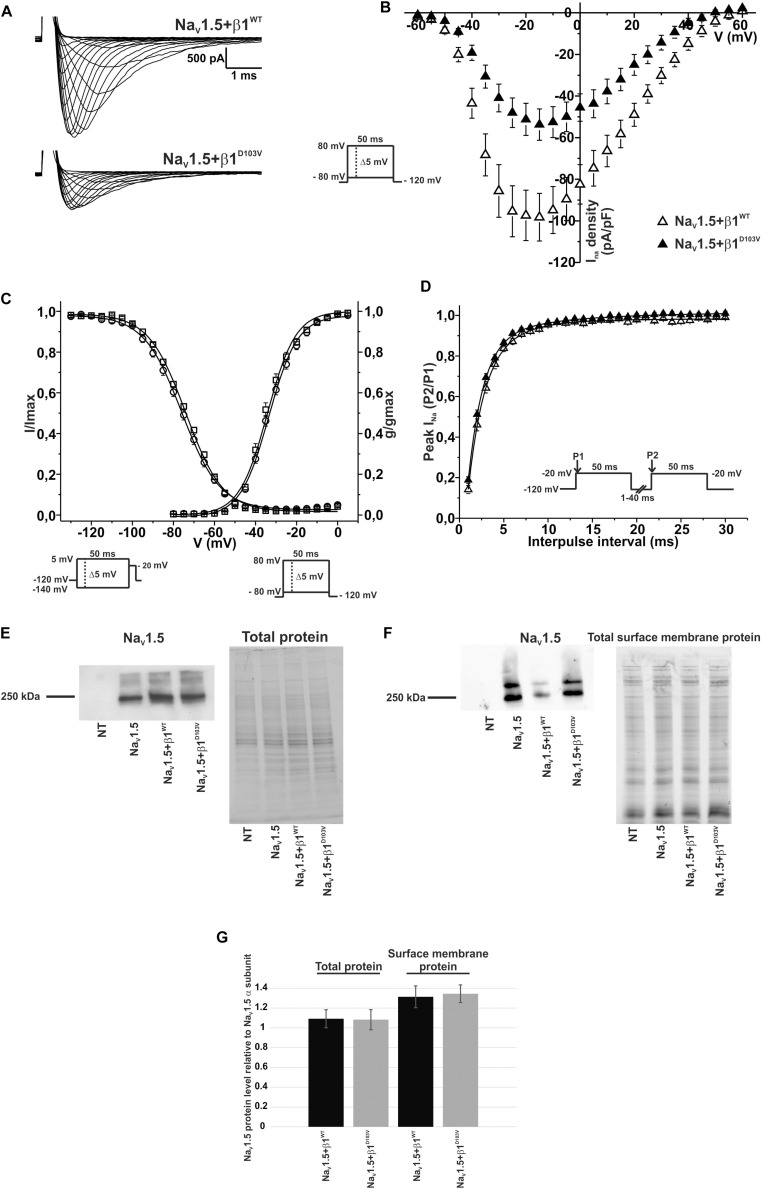
β1^D103V^ modifies the gating properties of Na_V_1.5 channel. Biophysical properties of sodium currents measured from HEK-293T cells expressing Na_V_1.5 alone, or co-expressed with either β1^WT^ or β1^D103V^. Whole cell currents were elicited by depolarizing potentials as shown in the insets. Open triangles are used to depict data for Na_V_1.5+β1^WT^ and filled triangles for Na_V_1.5+β1^D103V^. Solid lines in panels **(C)** and **(D)** represent the fitted curves. Values are expressed as mean ± SEM. **(A)** Representative whole-cell Na^+^ current traces from HEK-293T cells expressing Na_V_1.5+β1^WT^ (top), and Na_V_1.5+β1^D103V^ (bottom). **(B)** Mean current-voltage relationship. *I*_Na_ amplitude was normalized by the cell capacitance to obtain *I*_Na_ density values. **(C)** *I*_Na_ steady-state voltage dependence of activation and inactivation plots. **(D)** Recovery from inactivation curves. **(E)** Western blot detection of Na_V_1.5 (left) and corresponding total protein stain-free gel (right), from HEK-293T cells transfected with either Na_V_1.5, Na_V_1.5+β1^WT^, or Na_V_1.5+β1^D103V^ or non- transfected cells (NT) (*n* = 2). **(F)** Western blot detection of Na_V_1.5 after cell surface biotinylation (left) and corresponding total protein stain-free gel (right), from HEK-293T cells transfected with either Na_V_1.5, Na_V_1.5+β1^WT^, or Na_V_1.5+β1^D103V^ or non-transfected cells (NT). **(G)** Bar graph depicts the relative protein expression normalized by the Na_V_1.5 α subunit of total protein (left, *n* = 2) and biotinylated protein (right, *n* = 4) from HEK-293T cells transfected with either Na_V_1.5+β1^WT^ or Na_V_1.5+β1^D103V^. Both visible bands from the biotinylated samples were used for quantification, and the ratio was obtained by normalizing each condition with its respective input.

To determine whether β1^D103V^ affected the cardiac Na_V_1.5 sodium current, we co-expressed Na_V_1.5 with either the β1^WT^ or β1^D103V^ subunit (Figure 4A). Na_V_1.5+β1^D103V^ decreased sodium current density by 45.7% compared to Na_V_1.5+β1^WT^ (Figure 4B). β1^D103V^ induced a 3.37-mV positive shift of the voltage dependence of activation compared to β1^WT^. The voltage dependence of steady state inactivation was similar between Na_V_1.5+β1^WT^ and Na_V_1.5+β1^D103V^ (Figure 4C). Likewise, the recovery from inactivation time constants were similar when *I*_Na_ was measured in both experimental conditions (Figure 4D and Table 3).

**TABLE 3:** Biophysical parameters of HEK cells cotransfected with Na_V_1.5 and β1^WT^ or β1^D103V^.

	Peak *I*_Na_ density		Activation			Steady-state inactivation			Recovery	
	pA/pF	*n*	*V*_1/2_ (mV)	*k*	*n*	*V*_1/2_ (mV)	*k*	*n*	τ (ms)	*n*
Na_V_1.5+β1^WT^	−99.50 ± 12.05	13	−34.61 ± 0.68	6.40 ± 0.14	11	−74.90 ± 1.33	8.57 ± 0.25	11	2.27 ± 0.16	10
Na_V_1.5+β1^D103V^	−54.05 ± 7.63*	10	−31.24 ± 0.78*	6.52 ± 0.24	6	−74.76 ± 0.75	7.88 ± 0.25	10	2.14 ± 0.10	10

*Data is presented as Mean ± SE. I_Na_ = sodium current; n = number of cells; k = slope factor; V_1/2_ = voltage for half-maximal activation or steady-state inactivation; τ = time constant. ^∗^vs Na_V_1.5+β1^WT^. Significantly different, p-value < 0.05.*

Notably, we did not detect any sodium current in 34 of 75 cells transfected with Na_V_1.5+β1^D103V^. However, only 11 of 81 cells expressing Na_V_1.5+β1^WT^ had no detectable *I*_Na_. Nevertheless, β1 D103V mutation did not affect protein expression. We observed approximately the same amount of total Na_V_1.5 in all three conditions (Na_V_1.5 alone, Na_V_1.5+β1^WT^, and Na_V_1.5+β1^D103V^; Figures 4E,G). Moreover, the amount of Na_V_1.5 in the plasma membrane was unaffected by the β1 mutation. To determine this we performed immunoblotting analysis of biotinylated surface membrane proteins. Figures 4F,G show that the relative amount of plasma membrane Na_V_1.5+β1^WT^ and Na_V_1.5+β1^D103V^ channels was similar.

### The Mutant β1b^D103V^ Isoform Does Not Modify Na_V_1.5 Properties

**FIGURE 5:**
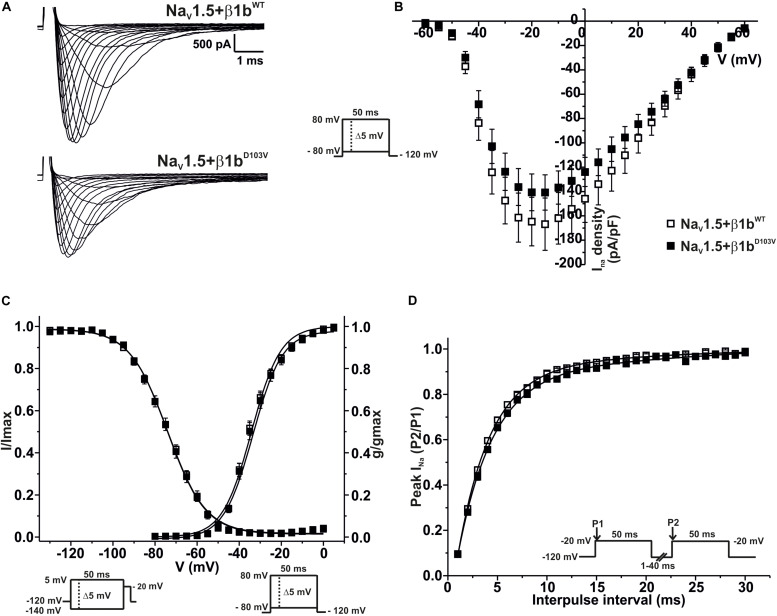
Na_V_1.5 current properties are not modified by β1b^D103V^. Biophysical properties of sodium currents measured from HEK-293T cells expressing Na_V_1.5 co-expressed with either β1b^WT^ or β1b^D103V^. Whole cell currents were elicited by depolarizing potentials as shown in the insets. Open squares are used to depict data for Na_V_1.5+β1^WT^ and filled squares for Na_V_1.5+β1^D103V^. Solid lines in panels **(C)** and **(D)** represent the fitted curves. Values are expressed as mean ± SEM. **(A)** Representative whole-cell Na^+^ current traces from HEK-293T cells expressing Na_V_1.5+β1b^WT^ (top), and Na_V_1.5+β1b^D103V^ (bottom). **(B)** Mean current-voltage relationship. *I*_*Na*_ amplitude was normalized to the cell capacitance to obtain *I*_Na_ density values. **(C)** *I*_*Na*_ steady-state voltage dependence of activation and inactivation plots. **(D)** Recovery from inactivation curves.

We finally evaluated the effects of β1b^D103V^ on Na_V_1.5 sodium current properties. The β1b^D103V^ mutation did not modify *I*_Na_ density compared to Na_V_1.5+β1b^WT^. Likewise, voltage dependence of activation, steady state inactivation, and recovery from inactivation were not altered by mutation compared to the wildtype subunit (Figure 5 and Table 4).

**TABLE 4:** Biophysical parameters of HEK cells cotransfected with Na_V_1.5 and β1b^WT^ or β1b^D103V^.

	Peak *I*_Na_ density		Activation			Steady-state inactivation			Recovery	
	pA/pF	*n*	*V*_1/2_ (mV)	*k*	*n*	*V*_1/2_ (mV)	*k*	*n*	τ (ms)	*n*
Na_V_1.5+β1b^WT^	−170.41 ± 22.22*	20	−34.69 ± 0.89	6.14 ± 0.28	20	−73.71 ± 0.62	9.25 ± 0.21	18	3.72 ± 0.11	10
Na_V_1.5+β1b^D103V^	−143.03 ± 14.84	14	−34.41 ± 1.15	6.29 ± 0.27	13	−74.21 ± 1.16	8.84 ± 0.20	10	4.15 ± 0.14	8

*Data is presented as Mean ± SE. I_Na_ = sodium current; n = number of cells; k = slope factor; V_1/2_ = voltage for half-maximal activation or steady-state inactivation; τ = time constant. ^∗^vs Na_V_1.5+β1b^WT^. Significantly different, p-value < 0.05.*

### The β1 Subunit Increases Na_V_1.1 Sodium Current Density

Because the proband also experienced brain pathologies, we investigated whether the D130V mutation in β1 and β1b isoforms also affected the most predominant brain-type sodium current: Na_V_1.1. We performed functional characterization of the sodium current in HEK-293T cells expressing Na_V_1.1 alone or co-expressed with β1^WT^ or β1b^WT^ subunits.

**FIGURE 6:**
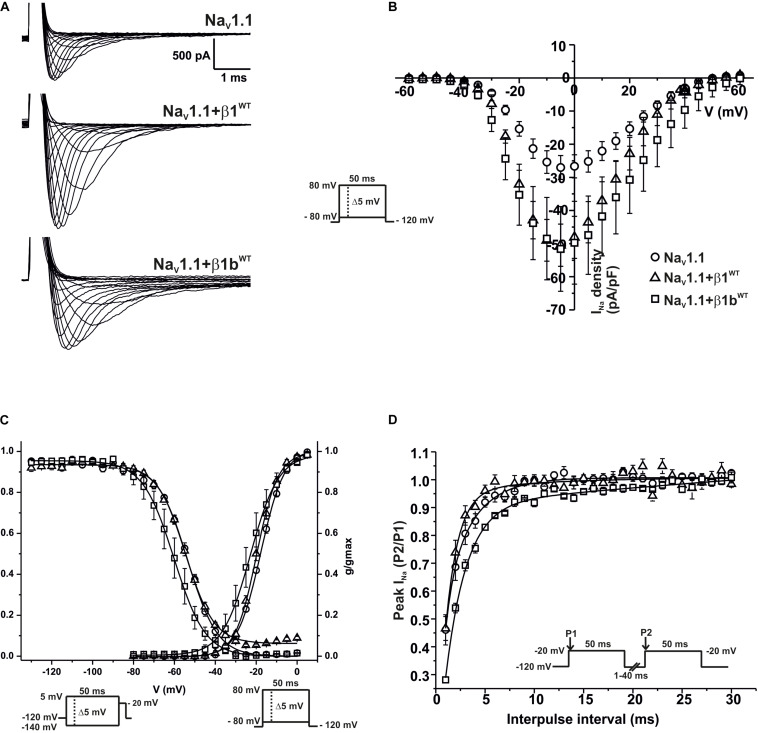
β1^WT^ and β1b^WT^ modulate Na_V_1.1 current. Biophysical properties of sodium currents measured from HEK-293T cells expressing Na_V_1.1 alone, or co-expressed with either β1^WT^ or β1b^WT^. Whole cell currents were elicited by depolarizing potentials as shown in the insets. Circles are used to depict data for Na_V_1.1 alone, triangles for Na_V_1.1+β1^WT^, and squares for Na_V_1.1+β1b^WT^. Solid lines in panels **(C)** and **(D)** represent the fitted curves. Values are expressed as mean ± SEM. **(A)** Representative whole-cell Na^+^ current traces from HEK-293T cells expressing Na_V_1.1 alone (top), Na_V_1.1+β1^WT^ (middle), and Na_V_1.1+β1b^WT^ (bottom). **(B)** Mean current-voltage relationship. *I*_Na_ amplitude was normalized by the cell capacitance to obtain *I*_Na_ density values. **(C)** *I*_Na_ steady-state voltage dependence of activation and inactivation plots. **(D)** Recovery from inactivation curves.

We measured macroscopic sodium currents (*I*_Na_) at varying potentials from these transfected cells (Figure 6A). There was a 45.9% increase of peak *I*_Na_ density when Na_V_1.1 was co-expressed with β1^WT^ compared to Na_V_1.1 alone. However, co-expression of Na_V_1.1 with β1b^WT^ did not significantly increase *I*_Na_ density compared to Na_V_1.1 alone (Figure 6B and Table 5). Neither β1^WT^ nor β1b^WT^ caused any significant changes in the voltage dependence of activation (Table 5). Steady-state inactivation of the Na_V_1.1 current was not altered by β1^WT^. On the contrary, co-expression of the β1b^WT^ isoform caused a 7.21 mV negative shift of the voltage dependence of inactivation compared to Na_V_1.1 alone (Figure 6C and Table 5). β1^WT^ markedly decreased the recovery from inactivation time in comparison to Na_V_1.1 alone. However, this decrease was not observed when Na_V_1.1 was co-expressed with β1b^WT^ (Figure 6D and Table 5).

**TABLE 5:** Biophysical parameters of Na_V_1.1 channels alone or cotransfected with β1^WT^ or β1b^WT^.

	Peak *I*_Na_ density		Activation			Steady-state inactivation			Recovery	
	pA/pF	*n*	V_1/2_ (mV)	*k*	*n*	*V*_1/2_ (mV)	*k*	*n*	τ (ms)	*n*
Na_V_1.1 alone	−27.39 ± 3.55	13	−17.71 ± 0.41	5.79 ± 0.19	7	−52.93 ± 0.80	7.21 ± 0.31	7	2.30 ± 0.12	6
Na_V_1.1+β1^WT^	−50.67 ± 6.38*	14	−19.69 ± 0.67	5.24 ± 0.14	9	−54.17 ± 0.73	7.28 ± 0.38	9	1.31 ± 0.26*	7
Na_V_1.1+β1b^WT^	−51.90 ± 12.75	5	−22.78 ± 2.91	6.05 ± 0.45	5	−60.14 ± 2.98*	6.75 ± 0.42	6	3.17 ± 0.52	5

*Data is presented as Mean ± SE. I_Na_ = sodium current; n = number of cells; k = slope factor; V_1/2_ = voltage for half-maximal activation or steady-state inactivation; τ = time constant. ^∗^vs Na_V_1.1. Significantly different, p-value < 0.05.*

### The β1^D103V^ Subunit Decreases Na_V_1.1 Current Density

**FIGURE 7:**
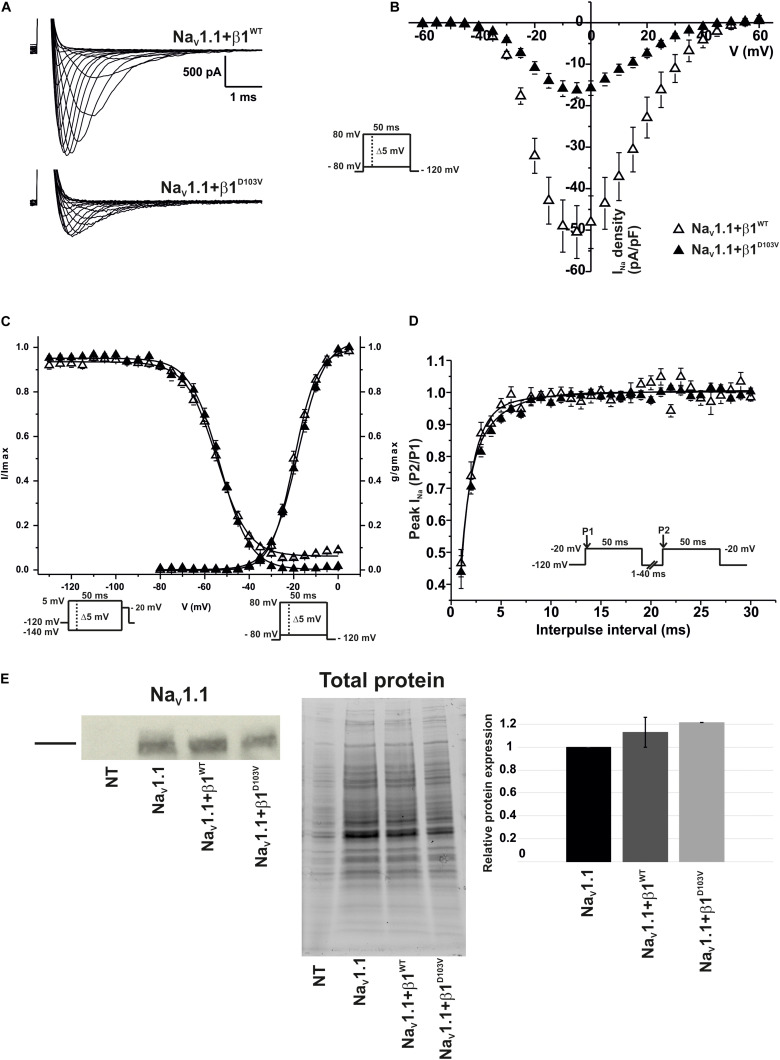
β1^D103V^ significantly decreases peak *I*_Na_ on Na_V_1.1. Biophysical properties of sodium currents measured from HEK-293T cells expressing Na_V_1.1 co-expressed with either β1^WT^ or β1^D103V^. Whole cell currents were elicited by depolarizing potentials as shown in the insets. Open triangles are used to depict data for Na_V_1.1+β1^WT^ and filled triangles for Na_V_1.1+β1^D103V^. Solid lines in panels **(C)** and **(D)** represent the fitted curves. Values are expressed as mean ± SEM. **(A)** Representative whole-cell Na^+^ current traces from HEK-293T cells expressing Na_V_1.1+β1^WT^ (top), and Na_V_1.1+β1^D103V^ (bottom). **(B)** Mean current-voltage relationship. *I*_Na_ amplitude was normalized by the cell capacitance to obtain *I*_Na_ density values. **(C)** *I*_Na_ steady-state voltage dependence of activation and inactivation plots. **(D)** Recovery from inactivation curves. **(E)** Western blot detection of Na_V_1.1 (left) and corresponding total protein stain-free gel (middle), from HEK-293T cells transfected with either Na_V_1.1, Na_V_1.1+β1^WT^, or Na_V_1.1+β1^D103V^ or non- transfected cells (NT). Bar graph on the right depicts the relative protein expression normalized by the Na_V_1.1 alone (*n* = 2).

To determine whether the β1 mutation identified in the patient had an effect on the neuronal Na_V_1.1 sodium current, we performed functional characterization in HEK-293T cells expressing Na_V_1.1+β1^D103V^ compared to those expressing Na_V_1.1+β1^WT^. Peak current density measured from cells expressing Na_V_1.1+β1^D103V^ was 66.9% smaller than that in Na_V_1.1+β1^WT^-expressing cells (Figures 7A,B and Table 6). Voltage dependence of activation and steady-state inactivation were not altered by β1^D103V^ (Figure 7C and Table 6). Recovery from inactivation time constants were similar in both experimental conditions (Figure 7D and Table 6).

**TABLE 6:** Biophysical parameters of Na_V_1.1 channels cotransfected with β1^WT^ or β1^D103V^.

	Peak *I*_Na_ density		Activation			Steady-state inactivation			Recovery	
	pA/pF	*n*	*V*_1/2_ (mV)	*K*	*n*	*V*_1/2_ (mV)	*k*	*n*	τ (ms)	*n*
Na_V_1.1+β1^WT^	−50.67 ± 6.38	14	−19.69 ± 0.67	5.24 ± 0.14	9	−54.17 ± 0.73	7.28 ± 0.38	9	1.31 ± 0.26	7
Na_V_1.1+β1^D103V^	−16.76 ± 1.88*	22	−18.18 ± 0.61	5.91 ± 0.21	13	−53.06 ± 0.34	7.09 ± 0.54	13	1.79 ± 0.18	7

*Data is presented as Mean ± SE. I_Na_ = sodium current; n = number of cells; k = slope factor; V_1/2_ = voltage for half-maximal activation or steady-state inactivation; τ = time constant. ^∗^vs Na_V_1.1+β1^WT^. Significantly different, p-value < 0.05.*

Similar to Na_V_1.5, we did not detect any current in 23 out of 59 cells expressing Na_V_1.1+β1^D103V^. However, when cells were transfected with Na_V_1.1+β1^WT^, only 4 of 56 cells showed no current. Western blot analysis showed no significant differences in Na_V_1.1 expression between the three conditions (Figure 7E).

### β1 Mutant Isoform b (β1b^D103V^) Modifies Na_V_1.1 Properties

**FIGURE 8:**
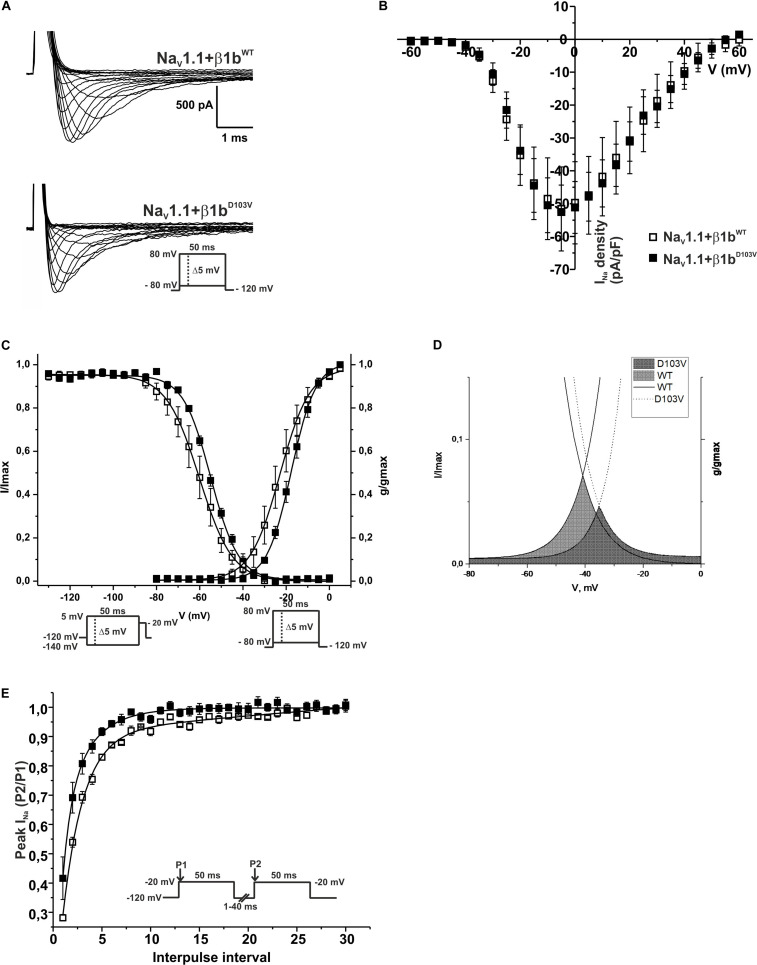
β1b^D103V^ modifies the gating properties of Na_V_1.1 channel. Open squares are used to depict data for Na_V_1.1+β1b^WT^ and filled squares for Na_V_1.1+β1b^D103V^. Solid lines represent the fitted curves. Values are expressed as mean ± SEM. Sodium currents were obtained with voltage clamp step protocols as shown in the insets. **(A)** Representative whole-cell Na^+^ current traces from HEK-293T cells expressing Na_V_1.1+β1b^WT^ (top), and Na_V_1.1+β1b^D103V^ (bottom). **(B)** Mean current-voltage relationship. *I*_Na_ amplitude was normalized by the cell capacitance to obtain *I*_Na_ density values. **(C)** *I*_Na_ steady-state voltage dependence of activation (right) and inactivation (left). **(D)** Window region bounded by the steady state activation and inactivation voltage dependent curves (Na_V_1.1+β1b^WT^ solid gray and Na_V_1.1+β1b^D103V^ diagonal pattern). **(E)** Recovery from inactivation curves.

Because the D103V mutation is located in a region shared by both β1 and β1b subunits, we assessed the effect of β1b^D103V^ on Na_V_1.1 current properties. We co-expressed Na_V_1.1 with either β1b^WT^ or β1b^D103V^ subunits (Figure 8A). Co-expression of β1b^D103V^ with Na_V_1.1 did not change *I*_Na_ density compared to Na_V_1.1+β1b^WT^ (Figure 8B and Table 7). Likewise, the β1b^D103V^ subunit did not significantly change the voltage dependence of activation compared to β1b^WT^. However, β1b^D103V^ caused a 6.04-mV right shift of the voltage dependence of steady-state inactivation when compared to the wildtype subunit (Figure 8C). This change resulted in a positive shift of the window current of Na_V_1.1+β1b^D103V^ compared to Na_V_1.1+β1b^WT^ (Figure 8D and Table 7). β1b^D103V^ significantly reduced the recovery from inactivation time constant compared to Na_V_1.1+β1b^WT^ (Figure 8E and Table 7).

**TABLE 7:** Biophysical parameters of Na_V_1.1 cotransfected with β1b^WT^ or β1b^D103V^.

	Peak *I*_Na_ density		Activation			Steady-state inactivation			Recovery	
	pA/pF	*n*	*V*_1/2_ (mV)	*k*	*n*	*V*_1/2_ (mV)	*k*	*n*	τ (ms)	*n*
Na_V_1.1+β1b^WT^	−51.90 ± 12.75	5	−22.78 ± 2.91	6.05 ± 0.45	5	−60.14 ± 2.98	6.75 ± 0.42	6	3.17 ± 0.52	5
Na_V_1.1+β1b^D103V^	−54.06 ± 8.41	9	−17.34 ± 1.16	5.36 ± 0.30	8	−54.10 ± 0.82*	6.36 ± 0.29	7	1.67 ± 0.23*	6

*Data is presented as Mean ± SE. I_Na_ = sodium current; n = number of cells; k = slope factor; V_1/2_ = voltage for half-maximal activation or steady-state inactivation; τ = time constant. ^∗^vs Na_V_1.1+β1b^WT^. Significantly different, p-value < 0.05.*

## Discussion

We present functional characterization of an *SCN1B* missense variant (c.308A>T; p.D103V) affecting both β1 and β1b regulatory subunits. This mutation was found in a newborn with heart and brain pathologies. The mutation was previously reported as pathogenic by Eldomery et al. (2017), potentially causing cardiomyopathies and intellectual disability among other phenotypes. The goal of our study was to determine whether the mutant β1^D103V^ and β1b^D103V^ subunits affected Na_V_1.5 and Na_V_1.1 sodium currents.

### Effects of β1^D103V^ but Not β1b^D103V^ Impair Normal Na_V_1.5 Function

HEK cells expressing Na_V_1.5+β1^WT^ showed increased *I*_Na_, consistent with Watanabe et al. (2008) and Qu et al. (1995). Also, we observed a shift of the voltage dependence of activation toward hyperpolarizing potentials in agreement with Ko et al. (2005) and Yuan et al. (2014). Lastly, we found a reduced recovery from inactivation time constant, consistent with Fahmi et al. (2001).

Mutations in the β1 subunit have been implicated in various cardiac arrhythmias, such as Brugada syndrome, atrial fibrillation, sudden infant death syndrome (SIDS), long QT syndrome, and cardiac conduction defect (Audenaert et al., 2003; Watanabe et al., 2008; Tan et al., 2010; Ricci et al., 2014; O’Malley and Isom, 2016). We show that the β1^D103V^ subunit does not increase *I*_Na_ as β1^WT^ does. In addition, the mutant subunit shifted the voltage dependence of activation toward more positive potentials, suggesting a physical interaction between β1^D103V^ and the alpha subunit. This interaction, however, appears not to disrupt Na_V_1.5 trafficking to the plasma membrane. Our data does not support this interpretation as relative plasma membrane protein levels are similar in both β1^WT^ and β1^D103V^ conditions. Thus the mutant β1 is likely to exert its effect over Na_V_1.5 is directly on the channel electrical properties. Taking into account that the β1 subunit is normally present in cardiac cells, this difference between β1^WT^ and β1^D103V^ would be considered a loss-of-function of the channel, consistent with the progressive atrial standstill and cardiac conduction disorder observed in the patient. It is necessary to point out that the patient is heterozygous for the β1 mutation. Thus, whether the mutation has a dominant negative effect remains to be determined. Further experiments in either patient-specific iPS derived cardiomyocytes or heterocygous heterologous expression would be needed to further explore this possibility.

The effect of the β1b^WT^ subunit on Na_V_1.5 current has not been well-studied. However, some groups have reported presence of the β1b^WT^ subunit in human and rat adult hearts, and expression levels of β1b in the atria and ventricle are greater than those of β1 (Kazen-Gillespie et al., 2000; Yuan et al., 2014). We observed an increase in Na_V_1.5 current density in the presence of β1b^WT^, consistent with previous works, as well as a decrease in the recovery from inactivation time constant. Although Na_V_1.5 current was diminished by the presence of the β1b^D103V^ subunit, acceleration in the recovery from inactivation was still observed with β1b^D103V^. This suggests that the interaction between Na_V_1.5 and the mutant β1b subunit is still present.

### Effects of β1^D103V^ on Na_V_1.1 Properties Could Impair Normal Neuronal Activity

Our results for neuronal Na_V_1.1 current show that β1^WT^ increases Na_V_1.1 I_Na_ density, in agreement with findings from Isom et al. (1992). In addition, we detected faster recovery from inactivation, as previously reported by Aman et al. (2009) and Barela (2006). Further, we show that the mutant β1 subunit strongly decreases Na_V_1.1 sodium current density compared to the wildtype β1 subunit, which represents a loss-of-function of the channel, in agreement with Meadows et al. (2002).

### β1b^D103V^ Has a Modulatory Effect Over Na_V_1.1 Function

The modulatory effect of the β1b subunit on Na_V_1.1 is poorly studied and controversial. Some groups propose that β1b expression predominates in embryonic development and early life and is thus essential for brain development (Kazen-Gillespie et al., 2000; Patino et al., 2011; O’Malley and Isom, 2016). Patino et al. (2011) studied an epilepsy-related mutation that only affects the β1b subunit. Although their co-immunoprecipitation studies did not detect an association between β1b and Na_V_1.1 or Na_V_1.3 channels, they showed that β1b modulates the Na_V_1.3 sodium current in heterologous systems. Our study shows that the β1b^WT^ subunit modulates voltage dependence of inactivation by shifting the curve to more negative potentials.

While β1b^D103V^ does not have any effects on Na_V_1.1 current density, we found that it shifts the voltage dependence of inactivation in the depolarizing direction, thus causing the channel to be available for activation in a larger voltage range. In addition, β1b^D103V^ strongly reduces the recovery from inactivation time constant compared to β1b^WT^. Thus, mutant channels are ready to be activated earlier than those without mutation. Assuming that β1b is normally present and interacts with Na_V_1.1 in native tissue, both effects would contribute to gain-of-function of the channel.

β1 subunit mutations that induce a gain-of-function of Na_V_1.1 have also been reported to cause brain dysfunction (Meadows et al., 2002; Kruger et al., 2016). Thus, it is possible that β1b^D103V^ causes neuron hyperexcitability, destabilizing normal neuronal behavior of the brain. Also, given the importance of this subunit to brain development (O’Malley and Isom, 2016), this mutation may impair brain formation at embryonic stages, thus provoking the brain phenotype at an early age, and may be involved in the patient’s polymicrogyria.

Our results showed that the modulatory effects of β1^D103V^ and β1b^D103V^ on Na_V_1.1 I_Na_ are different, even opposing each other. Considering that β1b is predominantly expressed during embryonic development, we expect that the gain-of-function effect of β1b^D103V^ is more important during that stage. A loss-of-function caused by β1^D103V^ would be more important later in development. Since the p.D103V mutation affects both β1 and β1b isoforms, it could differentially compromise neuronal electrical activity during development.

In conclusion, our results strongly suggest that the *SCN1B*_c.308A>T mutation contributes to the patient’s phenotype. We show that the β1^D103V^ mutant channels cause a loss-of-function of the cardiac-type sodium current, which could explain the clinical presentation of progressive atrial standstill, intra-atrial reentrant tachycardia, and cardiac conduction disorder in the child.

We surmise that the *SCN1B* variant could contribute to the patient’s brain phenotype at two different stages. During development and early life, when the β1b isoform is predominant, β1b^D103V^ leads to a gain-of-function of the channel. During adulthood, when the β1 subunit is predominantly expressed, β1^D103V^ produces loss-of-function of the sodium current. Both effects could contribute to the cognitive and motor deficits observed in the patient.

This family was part of a sequencing re-analysis project from which the *SCN1B* variant along with variants in *POLR1C* were flagged as the best candidates that potentially contribute to phenotypes manifested by both the proband and his elder sister. She had a fetal diagnosis of bradycardia and subsequent postnatal finding of complete AV block, leading to early neonatal death from multi-organ failure (Eldomery et al., 2017).

The proband’s father is heterozygous for the c.308A>T variant in *SCN1B* and for the c.88C>T variant in *POLR1C*, and his mother is heterozygous for the c.614delG variant in *POLR1C*. Both mother and father are asymptomatic. Incomplete penetrance is a common feature of channelopathies, and the idea of monogenetic disease has changed in recent years (Symonds and Zuberi, 2018). However, neither the *SCN1B* variant nor *POLR1C* variants found in the child could be the sole cause of his complex clinical picture.

*POLR1C* has been associated with autosomal recessive hypomyelinating leukodystrophy (Thiffault et al., 2015). This gene also has been described to cause recessive Treacher Collins syndrome 3 (OMIM #248390). Our proband did not show clinical manifestations of either of these conditions (Eldomery et al., 2017). However, the patient exhibited several neurological phenotypes consistent with *POLR1C* mutations, including hypomyelination, ataxia, and nystagmus, but the patient’s other clinical features are not described in *POLR1C*-related cases. Thus, it is possible that electrical disturbances caused by the *SCN1B* variant become more severe in the context of *POLR1C* mutations. Also, three-dimensional voltage mapping at baseline rhythm revealed extensive scarring of the right atrium and coronary sinus. Thus, electrical dysfunction potentially caused by the mutant β1 subunit could be aggravated by a damaged tissue substrate.

In summary, although the overall pathophysiology of the patient is complex, the Na_V_1.5 loss of function caused by the mutant β1 subunit D103V largely explains the clinical manifestations related to the patient’s heart dysfunction. In addition, loss of function of Na_V_1.1 caused by the β1 and β1b mutant subunits might aggravate a brain condition caused by the combination of the two *POLR1C* mutations.

## Data Availability Statement

The datasets generated for this study are available on request to the corresponding author.

## Ethics Statement

Written informed consent was obtained from the individual(s), and minor(s)’ legal guardian/next of kin, for the publication of any potentially identifiable images or data included in this article.

## Author Contributions

RM-M performed most of the experiments and analysis, made the figures, drafted the manuscript and contributed to the final version. ES revised all versions of the manuscript. HR performed the initial experiments and participated in the planning of the project. DC performed the cell surface protein biotinylation experiments. MP provided and supervised the neurology clinical aspects of the manuscript. CS provided and supervised the clinical cardiological aspects of the manuscript. MW provided the genetic data and participated in the planning of the project. GP and FS directed the project, supervised the experiments, and revised the data analysis and all versions of the manuscript. RB participated in the initial planning of the project and revised the manuscript. All authors contributed to manuscript revision, read and approved the submitted version.

## Conflict of Interest

The authors declare that the research was conducted in the absence of any commercial or financial relationships that could be construed as a potential conflict of interest.

## Footnotes

1 https://www.ncbi.nlm.nih.gov/clinvar/variation/375404

2 https://imagej.nih.gov/ij

3 https://imagej.nih.gov/ij
